# *Ferritin*, *N-Acetylated α-Linked Acidic Dipeptidase 2*, and *Cytoplasmic Aconitate Hydratase* Are Associated with Iron Metabolism and Regulate Iron Content in the Razor Clam, *Sinonovacula constricta*

**DOI:** 10.3390/ani16030441

**Published:** 2026-01-30

**Authors:** Ao Li, Zhihua Lin, Liyuan Lv, Hongqiang Xu, Hanhan Yao, Yinghui Dong

**Affiliations:** 1 College of Advanced Agricultural Sciences, Zhejiang Wanli University, Ningbo 315101, China; liaoliao25120@163.com; 2 College of Marine Life Sciences, Ocean University of China, Qingdao 266003, China; 3 Ninghai Institute of Mariculture Breeding and Seed Industry, Zhejiang Wanli University, Ninghai 315604, China; linzhihua@zwu.edu.cn (Z.L.); lvliyuan@zwu.edu.cn (L.L.); xuhongqiang@zwu.edu.cn (H.X.); 4 Zhejiang Key Laboratory of Aquatic Germplasm Resources, College of Biological & Environmental Sciences, Zhejiang Wanli University, Ningbo 315100, China

**Keywords:** *Sinonovacula constricta*, iron metabolism, *Ferritin*, *N-acetylated α-linked acidic dipeptidase 2*, *Cytoplasmic aconitate hydratase*

## Abstract

Iron is an important trace element, but there are few studies on iron metabolism in mollusks, and the key genes regulating iron content are still unclear. Our previous experiments have found that higher iron content was detected in the hepatopancreas and kidney of razor clam (*Sinonovacula constricta*). To clarify the key genes regulating the iron content in *S. constricta*, we focused on three candidates: *Ferritin* (*ScFER*), responsible for iron storage; *N-acetylated α-linked acidic dipeptidase 2* (*ScNAALAD2*), involved in iron transport; *Cytoplasmic aconitate hydratase* (*SccAH*), which may participate in the post-transcriptional regulation of *ScFER* and *ScNAALAD2*. Furthermore, their expression patterns and gene regulation under iron stress were further explored. When the external iron concentration increased, the iron content was at its highest levels in the hepatopancreas, and the expression of *ScNAALAD2* and *SccAH* decreased to the lowest levels, while the expression of *ScFER* increased. This suggests the possible transport and storage functions of these three genes in iron metabolism. In addition, after knocking down *SccAH*, the expression levels of *ScFER* increased, and the iron content also increased, further confirming the potential relationship between *ScFER* and iron content. Overall, we speculated that *ScFER* may play an important role in regulating the iron content of *S. constricta*. This study provides reference data for improving the iron metabolism pathway and exploring the main genes affecting iron content in mollusks.

## Simple Summary

Iron is a vital nutrient, but how mollusks manage iron in their bodies is not well understood. This study aimed to identify the key genes controlling iron levels in the razor clam. We focused on three genes, namely, *ScFER*, *ScNAALAD2*, and *SccAH*, involved in iron storage, transport, and regulation, respectively. When razor clams were exposed to more iron, the iron level rose in their hepatopancreas. At the same time, the activity of the storage gene increased, while the activities of the transport and regulatory genes decreased. Further experiments showed that reducing the activity of the regulatory gene led to higher activity of the storage gene and increased iron content. These results suggest that the iron storage gene *ScFER* likely plays a central role in controlling iron levels in the razor clam. This research helps us understand how mollusks process iron, which can inform strategies to improve mollusk nutrition and monitor environmental health.

## 1. Introduction

Iron is an essential trace element playing crucial roles in fundamental physiological processes including erythropoiesis, cell proliferation, and DNA synthesis [1]. Iron deficiency can lead to developmental impairments, weakened immunity, and cognitive deficits, posing a serious global health threat affecting approximately 25% of the population worldwide [1,2,3]. Improving the iron content of staple crops such as corn [4], rice [5], and cassava [6] through biotechnology is the primary strategy for food iron enhancement. But animal-derived iron-supplementing foods are also crucial to improving iron deficiency. The dynamic balance of iron in vivo is regulated by iron metabolism-related proteins, such as transferrin (Tf), transferrin receptor (TfRC), ferritin (FER), ferroportin (Fpn), divalent metal transporter 1 (DMT1), and N-acetylated α-linked acidic dipeptidase 2 (NAALAD2) that may exercise TfRC function in mollusks [7]. The iron responsive element (IRE) in the 5′ and 3′ untranslated regions (5′ UTR and 3′ UTR) of the corresponding mRNA of the above proteins binds to the iron-sensitive iron regulatory protein (IRP) to regulate its protein expression directly at the transcriptional or translational level. Cytoplasmic aconitate hydratase (cAH) is one of the bifunctional IRP, which is the IRP that binds to Fe^2+^ [8].

The razor clam *Sinonovacula constricta* is a traditionally important aquaculture mollusk in China. Crucially, it exhibits a notably higher iron content than other commonly cultivated species [9,10,11], highlighting its potential for the breeding of iron-rich varieties. Studies on iron metabolism in *S. constricta* have been reported. Li et al. [12] first proved that *FER* was involved in the regulation of iron metabolism and named it *ScFER*. Following its initial identification, *ScFER* has been shown to participate in immune defense [13]. Recombinant expression of ScFER further demonstrated its iron storage capacity, supporting its role in iron enrichment [14]. The crystal structure study has found that ScFER has structural similarity with vertebrate FER, offering a structural basis for understanding its iron uptake and storage mechanism [15]. Additionally, *FER* has been demonstrated to participate in iron metabolism of blood clam *Tegillarca granosa* [16] and yesso scallop *Patinopecten yessoensis* [17]. However, there has been limited research on the relationship between iron metabolism-related genes and iron content in mollusks, and the molecular mechanisms governing iron absorption, transport, and accumulation remain poorly understood. While plant studies have elucidated key molecular mechanisms of iron enrichment and demonstrated the efficacy of iron biofortification strategy [4,18,19], this concept remains unexplored in mollusks. Consequently, from a nutritional perspective, the breeding direction of high-quality mollusks mainly focuses on indicators such as glycogen and fatty acids, while the breeding of iron rich varieties has not yet been involved. Therefore, it is highly valuable to search for key genes that regulate the iron content and to stabilize iron enrichment as a heritable trait for breeding programs of razor clams.

This study thus aimed to identify and functionally assess key genes regulating iron content in *S. constricta*. Based on the genome, we focused on *ScFER*, *ScNAALAD2*, and *SccAH*, analyzing their characteristics and expression under iron stress and exploring their regulatory relationships via *SccAH* knockdown to pinpoint major genetic determinants of iron content. These results will advance the understanding of iron metabolism and offer insights for the genetic improvement of iron enrichment in razor clams. Specifically, the functional characterization of these genes lays a foundation for developing molecular markers to assist selection and provides potential targets for future genetic engineering approaches aimed at augmenting iron content.

## 2. Materials and Methods

### 2.1. Experimental Animals

In this study, 800 razor clams (one year old, the average shell length 6.58 ± 0.72 cm, the average body weight 19.74 ± 5.10 g) were obtained from Ningbo Ocean and Fishery Science and Technology Innovation Base (Ningbo, Zhejiang province, China). Prior to conducting the experiment, they were first cultured in ten tanks (50 cm × 40 cm × 25 cm) for 3 days. The temperature and pH were maintained at 25.0 ± 2.0 °C and 7.7–8.0, respectively. The seawater salinity of 20 ± 1 ppt (measured by water quality meter) was obtained by adding fresh water or sea crystal (Blue StarFish, Hangzhou, China) if necessary. The microalgae *Chlorella vulgaris* was fed twice a day at 6:00 and 18:00, and the culture water was continuously aerated and changed once a day. The water quality during the experiment was maintained at a level that did not result in mortality or observable stress behavior in razor clams. All experimental procedures were approved by the Institutional Animal Care and Use Committee of Zhejiang Wanli University, China.

### 2.2. Measurement of Iron Content

The different tissues of 30 razor clams, including gills, siphon, adductor muscle, foot, mantle, kidney, and hepatopancreas, were cleaned with normal saline. After freeze-drying (Labconco, 7522900, Kansas City, MO, USA), 30 individuals were divided into three groups, and ten individuals from each group were mixed into one sample pool. The samples were ground to powder with a mortar and then taken to determine the iron (Fe) content by inductively coupled plasma mass spectrometry (ICP-MS) (National Food Safety Standards of China, 2016f) [20].

To ensure data accuracy and precision, a comprehensive quality assurance and control protocol was implemented. The ICP-MS instrument was calibrated using a series of iron standard solutions, which yielded excellent linearity with correlation coefficients (R^2^) of 0.9996 (radial view) and 0.9991 (KED mode). The method demonstrated high sensitivity with limits of detection of 0.1645 ppm and 0.0713 µg/L for the respective modes. The protocol also included analysis of procedural blanks to monitor background contamination and duplicate analysis of samples to ensure methodological reproducibility. Recovery rates for iron were consistently maintained within 90–110% throughout the analytical batches.

### 2.3. Bioinformatic Analysis

The key genes, including *ScFER*, *ScNAALAD2* and *SccAH* were identified based on the published genome and transcriptome data of razor clams [21,22]. The open reading frames (ORFs) were predicted using NCBI ORF Finder (https://www.ncbi.nlm.nih.gov/orffinder/ (accessed on 5 March 2025)), while the signal peptide and conserved domains were identified with SignaIP-5.0 server (http://www.cbs.dtu.dk/services/SignalP/ (accessed on 13 March 2025)) and NCBI, respectively. Predicting transmembrane regions were predicted with SMART 10.0 (https://smart.embl.de/ (accessed on 7 February 2025)). RNA secondary structure was predicted using RNAfold web server 2.6.3 (http://rna.tbi.univie.ac.at//cgi-bin/RNAWebSuite/RNAfold.cgi (accessed on 25 February 2025)). The protein sequences of *FER*, *TfR*, *NAALAD2*, *IRP*, and *cAH* from various species were obtained via NCBI BLAST (https://blast.ncbi.nlm.nih.gov/Blast.cgi (accessed on 1 March 2025)), and multiple sequence alignments were analyzed by Jalview 9.0.5. The phylogenetic tree was constructed using the maximum-likelihood (ML) with the LG + I + G4 model using IQ-TREE 2.4.0, Boot-strapping with 1000 replications was performed to evaluate the phylogenetic tree, and the results were visualized by iTOL 7.4. Protein tertiary structure was predicted using AlphaFold Server 2.0 and visualized using PyMOL 3.1.3.

### 2.4. Iron Stress Treatment

A total of 162 razor clams were equally divided into the control and experimental groups, with the experimental group consisting of three replicates exposed to 2.8 mg/L FeSO_4_·7H_2_O [12] (Macklin, Shanghai, China). During the experiment, the feeding and other rearing conditions remained the same as those for acclimation (as described in Section 2.1), and half of the seawater was renewed at 17:00 each day. The tissues of gill, siphon, hepatopancreas, adductor muscle, and whole soft part were collected at 0 h, 3 h, 6 h, 12 h, 24 h, 48 h, and 72 h after iron exposure. At each time point, six individuals were selected from each group, and hepatopancreas were immediately frozen in liquid nitrogen and stored at −80 °C for iron content measurement.

### 2.5. Knockdown of SccAH Gene

**Figure A1:**
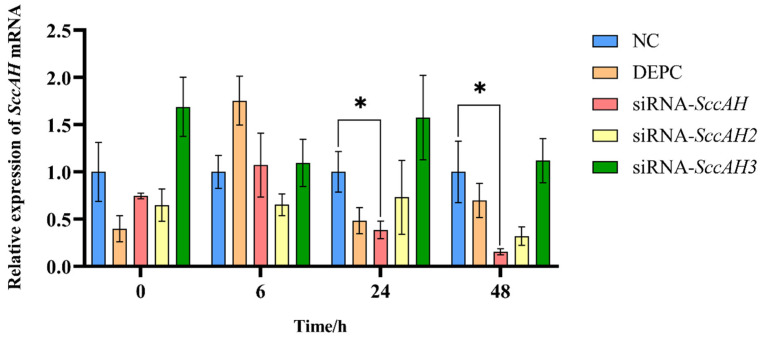
The pre-experiment results of siRNA-*SccAHs*. * indicates a significant difference (*p* < 0.05).

The *SccAH* gene was silenced using small interfering RNA (siRNA) (Sangon Biotech, Shanghai, China) targeting the *SccAH* mRNA. Three pairs of siRNAs primer sequences were designed based on the ORF sequence of *SccAH* gene (Appendix A Table A1). Following a preliminary experiment, the most effective siRNA chain (siRNA-*SccAH*) and optimal time point for achieving the highest interference efficiency were determined (Table 1, Appendix B Figure A1). A total of 240 healthy and non-injured razor clams were randomly divided into 3 groups: the experimental group (injected with siRNA targeting the *SccAH* gene), the control group (injected with DEPC water), and the negative control group (injected with NC siRNA). Each group was treated with an injection of 17.31 μL of siRNA (20 μM), DEPC-treated water, or NC siRNA (20 μM) into foot, respectively. After injection of siRNA, the razor clams were cultured in seawater, and the feeding and other rearing conditions remained the same as those for acclimation (as described in Section 2.1). At 0 h and 48 h after injection, six individuals from each group were selected, and their hepatopancreas tissues were dissected for gene expression and iron content analysis.

### 2.6. RNA Extraction and Quantitative Real-Time PCR (qRT-PCR)

Total RNA was extracted using total RNA extractor (Sangon Biotech, Shanghai, China), 1% agarose gel electrophoresis was used to detect RNA integrity, and ultramicro UV spectrophotometer (Implen, NanoPhotometer^®^, Munich, Germany) was used to determine RNA quality and concentration. cDNA was synthesized with the PrimeScript^®^ RT reagent kit (TaKaRa, Kyoto, Japan). Primers for *ScFER*, *ScNAALAD2*, and *SccAH* were designed using Oligo7 software, and *Ribosomal protein S9* (*RS9*) was used as the reference gene (Table 1) [23].

The qPCR primer pairs were rigorously validated prior to use. A standard curve was generated from a serial dilution of cDNA for each gene to determine amplification efficiency, which was confirmed to be between 90% and 105% with a correlation coefficient (R^2^) > 0.99. The specificity of each primer pair was confirmed by the presence of a single peak in the melt curve analysis and the amplification of a single band of the expected size on a 1% agarose gel. Then, qRT-PCR was performed with LightCycler^®^ 480 Instrument II (Roche, Basel, Switzerland) using SYBR qPCR master mix (Vazyme, Nanjing, China) in 20 μL reaction systems: 8 μL of cDNA (100-fold dilution), 1 μL of each primer, and 10 μL of SYBR mix. The amplification program included an initial denaturation at 95 °C for 10 min, followed by 40 cycles of 10 s at 95 °C and 60 s annealing at 58 °C. The gene relative expression levels were calculated using 2^−ΔΔCt^ method [24].

### 2.7. Analysis of ScFER Protein Expression

The polyclonal antibody anti-ScFER was produced according to the ScFER sequence by HuaBio (Hangzhou, China). The company confirms that all animal procedures are conducted in full compliance with national and institutional ethical guidelines for the care and use of laboratory animals. Anti-ScFER specificity was verified by Western blot. A single band at the expected molecular weight was observed, and no signal was detected when using pre-immune serum as a negative control, confirming the absence of non-specific binding.

Total protein was extracted from the hepatopancreas of razor clams subjected to iron stress (Section 2.4) and *SccAH* knockdown (Section 2.5) using high-efficiency RIPA buffer (Solarbio, Beijing, China), and quantified using PierceTM BCA protein assay kit (Thermo, Waltham, MA, USA). The protein was incubated with 5 × SDS-PAGE loading buffer at 98 °C for 10 min, separated by 12% SDS-PAGE, and transferred to a PVDF membrane (0.45 μm). After blocked with 5% non-fat powdered milk for 1.5 h, the PVDF membranes were incubated overnight at 4 °C with Anti-ScFER primary antibody (1:700 (*v*/*v*) dilution, initial concentration 8.28 mg/mL) (Hangzhou, China). The membranes were treated with corresponding HRP conjugate donkey anti-rabbit IgG (1:10,000 (*v*/*v*) dilution) (BBI, Shanghai, China) secondary antibody for 1 h at room temperature. In order to ensure accuracy and reliability of the results, the Anti-GADPH and Anti-TUBA4A (HRP conjugated) (1:1000 (*v*/*v*) dilution) (Sangon Biotech, Shanghai, China) were used as the reference proteins for iron stress and siRNA treatments, respectively, based on pre-experimental validation of their stability under each condition. Finally, the results were analyzed using ImageJ 2.14.0 software [25].

After iron stress (Section 2.4), fresh hepatopancreas of razor clams from the control and experiment groups at 0 h, 3 h, 6 h, 12 h, 24 h, 48 h, and 72 h after iron exposure were fixed with 4% paraformaldehyde, dehydrated by gradient ethanol, cleared in xylene/n-butanol, and finally paraffin-embedded sections (thickness 4 μm). Paraffin tissue sections were dewaxed with xylene, rehydrated with gradient ethanol, sealed with EDTA antigen repair solution for 1 h, and sealed with 5% BSA blocking solution. Rabbit polyclonal antibody against ScFER (1:500 (*v*/*v*) dilution) (HuaBio, China) was added and incubated at 4 °C overnight. The sections were incubated with FITC-labeled donkey anti-rabbit IgG secondary antibody (1:150, containing DAPI) (HuaBio, China) in the darkroom. Nikon 80i fluorescence microscope (Nikon, Tokyo, Japan) was used to observe and photograph the tissue immunofluorescence results.

### 2.8. Statistical Analysis

All data were shown as mean ± standard error (SE). The normality of data distribution was verified using the Shapiro–Wilk test, and the homogeneity of variances was confirmed using Levene’s test. One-way analysis of variance (ANOVA) followed by Duncan′s test was used to compare the difference in gene expression in various tissues and at different time points. Student′s *t*-test was used to compare the difference of iron content between the experimental and the control groups. The relationships between iron content and mRNA expression of *ScFER* and *ScNAALAD2* were assessed using linear regression and Pearson correlation analysis in GraphPad Prism 9.5. Assumptions of normality and homoscedasticity were verified prior to analysis. Statistical significance was defined as *p* < 0.05 for all tests.

## 3. Results

### 3.1. Sequence and Structure Analysis of ScFER, SccAH, and ScNAALAD2

**Figure 1:**
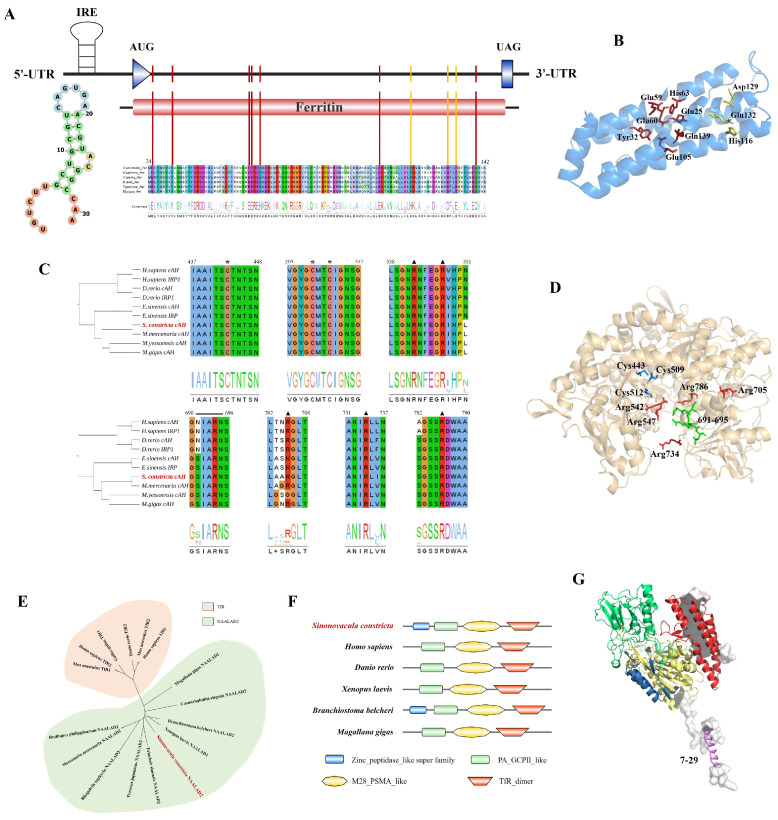
Sequence and structure analysis of ScFER, SccAH, and ScNAALAD2. (**A**) Sequence analysis of ScFER. The denote ferrous ion oxidase sites and iron ion access channel were marked in the red and yellow vertical lines, respectively. (**B**) The tertiary structure of ScFER. The sites marked in red and yellow represent ferrous ion oxidase sites and iron ion access channel, respectively. (**C**) Homologous sequence alignment and phylogenetic tree of SccAH. The asterisk and triangulated positions are cysteine and arginine, respectively, which are indispensable for the assembly of iron–sulfur clusters. The amino acids necessary for the recognition of IRE are marked with double horizontal lines. (**D**) The tertiary structure of SccAH. The blue, red, and green regions correspond to the positions of the marked asterisks, triangles, and double horizontal lines in (**C**), respectively. (**E**) Phylogenetic tree of ScNAALAD2. (**F**) Comparison of protein domains of TfR and NAALAD2. (**G**) The tertiary structure of ScNAALAD2. The color of each domain corresponds to (**F**), and the amino acids at 7–29 positions marked by pink are transmembrane regions.

The ORF of *ScFER* (GenBank accession number: GQ906972) consisted of 516 bp encoding a 171-amino acid with an average molecular mass of 19.72 kDa and pI value of 4.952, without signal peptide and transmembrane region, and contained a eukaryotic ferritin domain. Blastp analysis showed that the similarity between ScFER and other species was high, and the identity was 50.90–84.02%. The similarity of ScFER with *Homo sapiens* Ferritin heavy chain (FTH) (NP_002023) (66.47%) was higher than that of *H. sapiens* Ferritin Light Chain (FTL) (CAG32996) (50.90%), which indicated that ScFER was an H-type ferritin. By sequence alignment, an *IRE* was identified in the 5′ UTR of *ScFER*. The IRE sequence folded into a typical stem-loop structure containing CAGUGA (Figure 1A), which regulates ferritin synthesis at the translation level. Two functional sites were identified, including ferrous ion oxidase site and iron ion access channel. The iron oxidase center was located at the center of ScFER, consisting of Glu25, Tyr32, Glu59, Glu60, His63, Glu105, and Gln139. The entry and exit channels for iron ions are composed of His116, Asp129, and Glu132 (Figure 1B).

Phylogenetic tree analysis and sequence alignment revealed that IRP1 and cAH from the same species are highly conserved and clustered together as the first group (Figure 1C). Blastp analysis showed that SccAH (GenBank accession number: PV963841) shared high similarity with other species, ranging from 53.96 to 93.44%. At conserved positions across the ten sequences, both cAH and IRP1 exhibited all the amino acid residues required for iron-sulphur cluster assembly and two mutually exclusive activities of IRP1. This included three cysteine residues (Cys443, Cys509, Cys512) essential for iron–sulfur cluster binding (Figure 1C, asterisk), four arginines involved in substrate binding (Arg542, Arg547, Arg705, Arg786), three of which (Arg542, Arg547, Arg786) were involved in IRE binding, Arg734, which interacts with IRE bulges (Figure 1C, triangulation), and a region containing residues 691–695, necessary for IRE loop recognition (Figure 1C, double horizontal line). These sequences may play a key role in maintaining the tertiary structure of IRP1 and the spatial conformation of IRE/IRP1 complexes (Figure 1D).

ScNAALAD2 (GenBank accession number: PV963842) contains a signal peptide and transmembrane region (Figure 1G), along with conserved domains similar to vertebrate TfR (Figure 1F), including M28_PSMA_like domain, PA_GCPII_like domain, and TfR_dimer domain. Blastp analysis revealed that ScNAALAD2 shared the highest consistency with *Branchiostoma belcheri* (XP_019647645), with a similarity of 43.57%. Among the TfR family members, NAALAD2 was most closely related to TfR. The phylogenetic tree showed that ScNAALAD2 clustered with NAALAD2 from other species, followed by vertebrate TfR (Figure 1E).

### 3.2. Changes in Iron Content and Expression of Iron Metabolism Genes Under Iron Stress

**Figure 2:**
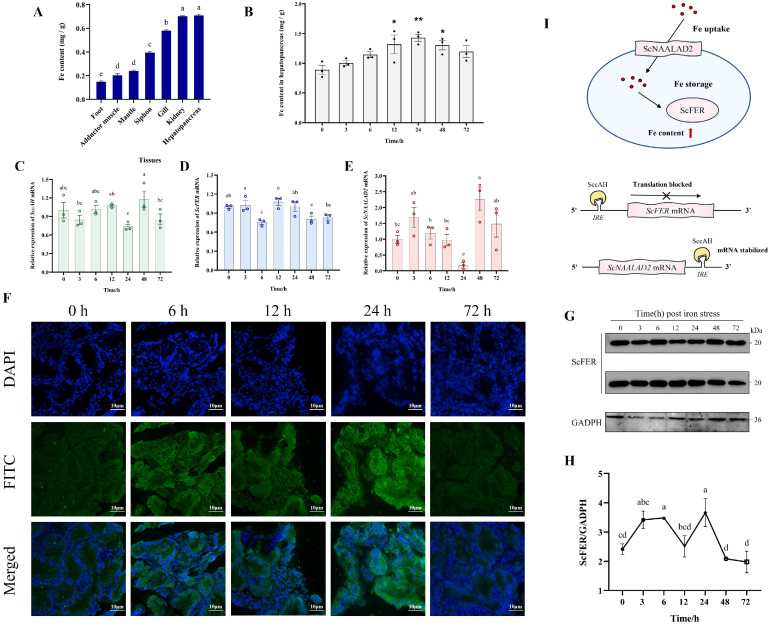
Iron content and related gene/protein expression patterns under iron stress. This figure demonstrates the temporal dynamics of iron accumulation and the responsive expression of key genes in the hepatopancreas of *S. constricta* following iron exposure. (**A**) Iron content in seven different tissues prior to exposure. The change of iron content (**B**) and the expression profiles of *SccAH* (**C**), *ScFER* (**D**) and *ScNAALAD2* (**E**) in hepatopancreas under iron stress. (**F**) Expressions of ScFER protein in the hepatopancreas after iron stress by immunofluorescence staining. Scale bars = 10 μm. (**G**, **H**) Expression of ScFER protein in the hepatopancreas by Western blot. Detection (**G**) and quantitation analysis (**H**) of the relative expression of ScFER with stress time progression. GAPDH was used as an internal control. (**I**) Speculated iron metabolism diagram of *S. constricta*. The red arrows indicate an increase in iron content Asterisks represent significant difference between iron stress and control (0 h) groups. (* *p* < 0.05, ** *p* < 0.01). Different lowercase letters indicate the significant difference among groups (*n* = 3).

The iron content of seven tissues in *S. constricta* was determined. The results showed that the iron content in the hepatopancreas and kidney were both higher than other tissues. The hepatopancreas, as a major iron storage organ, contained substantially more iron content than the gill and siphon. The tissues with the lower iron content were mantle, adductor muscle, and foot (Figure 2A).

Under exposure to 2.8 mg/L FeSO_4_·7H_2_O, the iron content in the hepatopancreas increased, peaked at 24 h and then decreased (Figure 2B). The expression levels of *ScFER* mRNA increased from 0 to 3 h, decreased to a minimum at 6 h, and peaked again at 12 h before declining thereafter (Figure 2D). In contrast, *SccAH* mRNA expression levels decreased from 0 to 3 h, then gradually increased to a peak at 12 h, fell to its lowest level at 24 h, rose again from 24 to 48 h, and subsequently decreased (Figure 2C).The expression level of *ScNAALAD2* declined sharply after 3 h and reached the lowest at 24 h, then gradually increased to a peak at 48 h, followed by a subsequent decline through 72 h (Figure 2E). Notably, the iron content in the hepatopancreas peaked at 24 h, significantly exceeding the control level at 0 h (*p* < 0.01) (Figure 2B). At this point, the expression levels of *SccAH* and *ScNAALAD2* were the lowest, while *ScFER* remained at a relatively high level (Figure 2C–E). Western blot analysis showed that ScFER protein expression increased from 0 to 6 h, decreased from 6 to 12 h, increased again after 12 h, and then declined continuously after 24 h in the hepatopancreas (Figure 2G, H). Notably, the expression level of *ScFER* at 24 h was significantly higher compared to 0 h (*p* < 0.05). The expression levels of *ScFER* and the iron content both reached their highest levels at 24 h in the hepatopancreas. The green fluorescent signal exhibited the location of ScFER protein, which was mainly expressed in the cytoplasm (Figure 2F). Based on these findings, a schematic model summarizing the potential iron metabolism pathways in *S. constricta* was proposed (Figure 2I).

### 3.3. Changes in SccAH and ScFER Expression and Iron Content After siRNA Treatment

**Figure 3:**
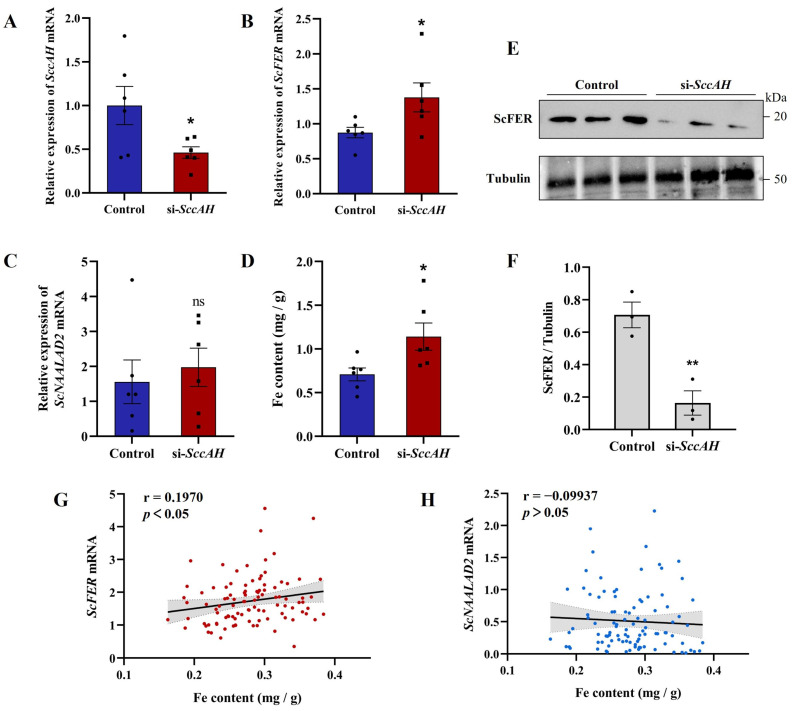
Effect of *SccAH* knockdown on iron homeostasis and related gene/protein expressions in *S. constricta* hepatopancreas. (**A**) The relative expression of *SccAH* after interference (*n* = 6). (**B**) The relative expression of *ScFER* after knockdown of *SccAH* (*n* = 6). (**C**) The expression level of *ScNAALAD2* after knockdown of *SccAH* (*n* = 6). (**D**) Fe content changes after knockdown of *SccAH* (*n* = 6). Detection (**E**) and quantitation analysis (**F**) of the relative expression of ScFER protein in the hepatopancreas after knocking down *SccAH* by Western blot (*n* = 3). Tubulin alpha 4a was used as an internal control. (**G**) Correlation analysis between iron content and the mRNA expression levels of *ScFER*. (**H**) Correlation analysis between iron content and the mRNA expression levels of *ScNAALAD2*. The vertical bars represent the mean ± S.E, * *p* < 0.05, ** *p* < 0.01, ns means no significant difference.

Following siRNA treatment, the expression levels of *SccAH* were significantly decreased compared to the control group in the hepatopancreas (*p* < 0.05) (Figure 3A). Meanwhile, *ScFER* expression levels increased significantly in the interference group compared to the control group (*p* < 0.05) (Figure 3B), while no significant difference in *ScNAALAD2* expression was observed between the interference and control groups (*p* > 0.05) (Figure 3C). Consistent with these trends, the iron content in the *SccAH* interference group was significantly higher than the control group (*p* < 0.05) (Figure 3D). To further investigate the regulatory mechanism of *SccAH* on *ScFER*, the expression levels of ScFER protein were further analyzed in the interference and control groups. The results showed a decrease in ScFER protein expression in the interference group (Figure 3E,F). Compared with the control group, the *ScFER* expressions at the transcription and translation levels exhibited an opposite trend.

A correlation analysis between iron content and the expression levels of *ScFER* and *ScNAALAD2* was conducted in 100 razor clams. The results showed that there was a positive association between *ScFER* expression levels and iron content, but the correlation was weak (*p* = 0.0428, r = 0.1970) (Figure 3G). However, there was no significant correlation between *ScNAALAD2* expression and iron content in razor clams (*p* = 0.5776, r = −0.09937) (Figure 3H).

## 4. Discussion

This study explored the key genes influencing the iron metabolism in razor clams. The results revealed that razor clams exhibited higher iron content in the hepatopancreas and kidney compared to other tissues, and *ScFER* and *SccAH* were identified as potential key regulators of iron content. These findings not only provide insights into the molecular mechanisms of iron metabolism but also support iron-enriched variety breeding in razor clams.

FER is the main iron storage protein, which is widely found in plants, animals and microorganisms. ScFER was highly homologous to the known FTH and belonged to H-type ferritin, and clustered more closely with other mollusks than other species, which was consistent with the conclusion of Li et al. [12]. In marine mollusks, FER is the main iron storage protein and plays a role in iron storage, release, and transport [26]. Sequence analysis revealed a canonical *IRE* in the 5′ UTR of *ScFER*, forming a hairpin structure composed of stem loops, with a typical *IRE* loop sequence CAGUGA. *IRP1* binds to the *IRE* and inhibits translation by preventing the binding of small ribosomal subunits to the mRNA, thereby regulating *FER* expression at the post-transcriptional level [27]. *IREs* are present in almost all *FER* mRNAs, which has been confirmed in some species, such as the amphioxus *B. belcheri* [28], *P. yessoensis* [17], and the freshwater giant prawn *Macrobrachium rosenbergii* [29]. *IREs* also exist in the untranslated regions of many genes involved in iron metabolism, such as *TfR* and *DMT1*, and play roles in protecting transcripts or blocking translation [30]. Furthermore, both sequence alignment and tertiary structure prediction confirmed that the iron oxidase center sequence of ScFER was highly conserved and located at the center of the ferritin cage structure, representing a typical functional feature of ferritin in most marine invertebrates [15].

*IRP1* is a bifunctional protein. Under conditions of low intracellular iron concentration, *IRP1* binds to *IREs* and inhibits the translation of target genes, such as *FER*, thereby releasing stored iron for physiological activities [31]. In contrast, when the intracellular iron level is high, *IRP1* assembles an aconitase-type iron–sulfur cluster, thereby changing the conformation of *IRP1*, losing its binding ability to *IRE*, and acquiring enzymatic activity as *cAH*, which plays a catalytic role in the cytoplasm [32]. SccAH is highly homologous to IRP1, and contains amino acid residues (Cys443, Cys509, Cys512) necessary for the assembly of iron–sulfur clusters, as well as sequences (Arg542, Arg547, Arg705, Arg786, and the 691–695 region) essential for identifying and binding IRE. These sequences play a key role in maintaining the three-dimensional structure of IRP1, which suggests that it may perform the same function as IRP1 when its iron–sulfur cluster is decomposed.

*TfR* mediates cellular uptake of *Tf* and H-type *FER*, and its expression is regulated by cellular iron status [33]. The *TfR* family has an ancient homologous protein, *NAALAD2* in *S. constricta* (*ScNAALAD2*). In evolution, *NAALAD2* appeared before *TfR* [34]. *ScNAALAD2* had domains that were consistent with the *TfR* of higher vertebrates, namely, the dimerization (helical) and peptidase domains. *ScNAALAD2* also contained a signal peptide and a transmembrane region. In white sea cold-water sponges *Halichondria panicea* and *Halisarca dujardini*, *NAALAD2* may replace *TfR* to participate in iron metabolism [35]. Therefore, it is speculated that *ScNAALAD2* may have both enzymatic and binding abilities and act as a carrier of Fe^3+^ in iron metabolism, exerting an iron transport function consistent with *TfR* [7].

In vertebrates, the liver serves as the primary organ for iron storage and systemic iron homeostasis, while in invertebrates such as bivalves, this role is assumed by the hepatopancreas [1,36,37]. In the present study, we measured the iron content in seven tissues of *S. constricta* and obtained the consistent results. The hepatopancreas (liver) and kidney had the highest iron content, and iron content of the hepatopancreas was significantly higher than the kidney, indicating its important roles in regulating iron metabolism and maintaining iron homeostasis in *S. constricta.*

*ScNAALAD2*, as a member of the TfR family, is a homolog of the transferrin receptor *TfR* and contains dimerization (helical) and peptidase domains. It may have enzymatic function and ability to mediate iron uptake, and can be used as a carrier of Fe^3+^ [7]. There have been a lot of studies on *TfR* in vertebrates, and it mainly mediates cellular iron uptake by binding to *Tf*. Iron deficiency occurs when the mammalian red blood cell *TfR* cycle is impaired [38]. Our results also showed that *ScNAALAD2* reached its lowest expression level after 24 h of iron stress, while the iron content of the hepatopancreas was the highest at this time, which suggested that the *TfR* may be involved in the regulation of iron content. After 6 h, the expression of *ScNAALAD2* continued to decrease. The body reduced iron intake by downregulating *ScNAALAD2* expression and increased *ScFER* expression to store excess iron, thereby preventing iron overload. However, the results of the correlation analysis showed no correlation between *ScNAALAD2* expressions and iron content in the whole soft tissue of *S. constricta* (*p* > 0.05, r = −0.09937). This suggests that *ScNAALAD2* may be involved in iron metabolism regulation by affecting cellular iron uptake rates, but does not directly determine total iron content [39].

*SccAH* is an *IRP1* in the iron–sulfur cluster decomposition state [40], which may function as IRP. The binding activity of *IRP1* to *IRE* located on *FER* mRNA, and the content of non-heme iron in liver were negatively correlated [41]. In the IRP-IRE signaling pathway, the increase level of *FER* is achieved by the reduction in *IRP* [42], which was consistent with our study. When the iron content increased to the highest level from 12 h to 24 h, the expression level of *SccAH* showed a downward trend, and *SccAH* and *ScFER* exhibited a consistent fluctuation trend during iron stress. Nadimpalli et al. [31] also found that the iron levels were lower when *IRP1* activity was higher. It is worth noting that at 24 h of iron stress, the iron content of hepatopancreas reached the peak, which was significantly higher than the control group (0 h). At this time, *ScNAALAD2* and *SccAH* were at the lowest expression level. On the contrary, although *ScFER* expression showed a downward trend after peaking, it remained relatively high throughout 0 h–72 h. This observation is consistent with the hypothesis that *ScNAALAD2* may act as a putative transporter involved in iron uptake, while *ScFER* functions in iron storage. Cytoplasmic ferritin resists ferroptosis by controlling iron availability [43]. In this study, the trend of *ScFER* expression from 0 h to 24 h was consistent with that reported by Li et al. [12], indicating that the expression of *ScFER* can be induced by iron treatment and involved in iron storage in *S. constricta*. Although we have analyzed expression changes in *ScFER*, *ScNAALAD2* and *SccAH* after iron stress, a variety of physiological indicators still need to be measured to rule out potential toxic effects in the future.

Moreover, the correlation analysis showed that the increase in *ScFER* expression corresponded to an increase of iron content (*p* < 0.05, r = 0.1970). Similarly, in zebrafish, the low expression of ferritin was found to correspond to a reduction in cellular and mitochondrial iron levels, which also indicated a positive regulatory relationship between ferritin and iron content [44]. In extracellular vesicles, it was also found that H-type ferritin increased significantly in an iron-loaded state [27]. These studies have indicated that the expression level of *ScFER* increases, along with its iron storage function, leads to an increase of iron content. In addition, the correlation analysis used the iron content of the whole soft part, which may be one of the reasons for the weak correlation. In addition, ScFER protein expression and iron content both exhibited an upward trend during 12 h–24 h after iron stress, indicating that the translation efficiency or protein content of ferritin may have a stronger positive correlation with iron content.

In organisms, iron homeostasis is primarily regulated through the binding of IRP to *IRE* in target mRNA, thereby controlling their translation and stability [45]. IRP is a bifunctional protein that plays a key role in regulating *FER* mRNA. When assembling iron–sulfur clusters, it changes its conformation and transforms into cAH to perform function [32]. In the present study, *SccAH* was knocked down to explore its effects on iron content and regulatory relationships with *ScFER* and *ScNAALAD2*. After *SccAH* knockdown, the mRNA expression levels of *ScFER* increased, and the iron content also increased accordingly. Santamaria et al. [46] inactivated *IRP1* using oxalomalate and observed increased *FER* mRNA levels, which was consistent with our findings. This phenomenon may be due to the increased stability of *FER* mRNA. In *IRP2* knockout mice, liver iron accumulation was also observed [47]. The observed positive correlation between tissue iron content and *ScFER* mRNA levels aligns with findings in vertebrates, where the liver iron content increasing is associated with increased *FER* expression and decreased *TfR1* expression [48]. Related studies have confirmed the iron storage function of ScFER: iron can enter the internal cavity of ScFER through the iron ion channel located on ScFER, and then stored in the internal cavity of ScFER [15].

However, there was no translation activation of *ScFER* in the present study. On the contrary, the expression of ScFER protein level showed a downward trend compared with the control group. In *IRP1* knockout mice, the translation efficiency of *FTL1* and *FTH1* exhibited an upward trend, and *FTH1* increased significantly, but there was no significant difference compared with the control group. Upon *IRP2* knockout in mice, *FER* mRNA translation exhibited distinct effects under different light cycles: it was strongly inhibited during the daytime light phase and remained low during the nighttime dark phase, respectively [31]. Combined with the decrease expressions of *ScFER* observed in this study, we propose that this may be related to our sampling occurring during the daytime. It is possible that specific repressors are active in the light phase, which could inhibit *ScFER* translation. During iron depletion, FER is transported into the bloodstream from cells, thereby leading to its protein level decreasing in tissues [48]. This may explain the phenomenon of ScFER protein expression decreasing and tissue iron content increasing in razor clams. In mammals, the increase of iron export caused by the upregulation of membrane iron transporter *Fpn* may explain reduced *FER* levels [49]. Reduced *IRP* activity and decreased expression levels of *TfR* and *FER* were found in scrapie-infected mouse neuroblastoma N2a cells [50], which suggested that *FER* expression was also modulated by iron-independent signaling pathways related to inflammation and stress [51,52]. However, the potential roles of circadian regulation, iron export, and tissue-specific homeostasis remain speculative in razor clams. Future work is needed to test these hypotheses and to fully elucidate the involved molecular networks.

## 5. Conclusions

In summary, the hepatopancreas was the main iron storage tissue in razor clams. The key genes related to iron metabolism, including *ScFER*, *ScNAALAD2*, and *SccAH* were identified, and their expression patterns under iron stress were further explored. After iron stress, the expression level of *ScNAALAD2* was the lowest, leading to a decrease of iron transport. In contrast, the expression of *ScFER* increased, resulting in enhanced iron storage, and the iron content in the hepatopancreas reached its highest level. In addition, after *SccAH* knockdown, the expression of *ScFER* increased with the increase of iron content in hepatopancreas, suggesting the regulation of *SccAH* on *ScFER* and its iron storage function. These preliminary findings provide evidence for the potential role of *ScFER* in regulating tissue iron content in razor clams. However, the precise molecular mechanisms and interactions that regulate the whole soft part iron content remain to be fully elucidated. This study provides a preliminary theoretical foundation for exploring the key genes involved in iron metabolism and enrichment in razor clams.

## Figures and Tables

**Table 1:** Primer sequences of the key genes.

Primer Name	Sequence	GenBank Number	Primer Efficiency Value	Amplicon Size (bp)	Application
siRNA-SccAH-F	GAGUCAUACUGCAAGACUUTT	-	-	-	RNAi
siRNA-SccAH-R	AAGUCUUGCAGUAUGACUCTT				
NC-F	UUCUCCGAACGUGUCACGUTT	-	-	-	
NC-R	ACGUGACACGUUCGGAGAATT				
SccAH-F	GGTGCCGATAGTGTTTGATG	PV963841	99.87%	101	qRT-PCR
SccAH-R	TGACCGATGGATTGCTTG				
ScFER-F	ATTGTCCTCCAACCCATCTC	GQ906972	99.77%	196	
ScFER-R	TGGCTTCCACTTGCTCCTC				
ScNAALAD2-F	CAGTGCTCTTTGTGGTCGTT	PV963842	99.86%	190	
ScNAALAD2-R	CGTGGCTTGCTCGTGTAGTT				
RS9-F	TGAAGTCTGGCGTGTCAAGT	OQ244850	99.84%	117	Reference gene
RS9-R	CGTCTCAAAAGGGCATTACC				

*ScFER* (Ferritin), *ScNAALAD2* (N-acetylated α-linked acidic dipeptidase 2), *SccAH* (Cytoplasmic aconitate hydratase), *RS9* (Ribosomal protein S9).

## Data Availability

All the datasets in this study can be provided upon reasonable request.

## Abbreviations

The following abbreviations are used in this manuscript:

Tf	Transferrin
TfRC	Transferrin receptor
FER	Ferritin
Fpn	Ferroportin
DMT1	Divalent metal transporter 1
IRE	Iron responsive element
NAALAD2	N-acetylated α-linked acidic dipeptidase 2
cAH	Cytoplasmic aconitate hydratase

## Appendix A

**Table A1:** siRNAs primer sequences used in the experiment.

Primer Name	Sequence
siRNA-SccAH-F	GAGUCAUACUGCAAGACUUTT
siRNA-SccAH-R	AAGUCUUGCAGUAUGACUCTT
siRNA-SccAH2-F	UUGAAGAACAUGCUGAUUGTT
siRNA-SccAH2-R	CAAUCAGCAUGUUCUUCAATT
siRNA-SccAH3-F	CUGACAUUGUUCUCACUAUTT
siRNA-SccAH3-R	AUAGUGAGAACAAUGUCAGTT
